# Pre-graduation medical training including virtual reality during COVID-19 pandemic: a report on students’ perception

**DOI:** 10.1186/s12909-020-02245-8

**Published:** 2020-09-25

**Authors:** Roberto De Ponti, Jacopo Marazzato, Andrea M. Maresca, Francesca Rovera, Giulio Carcano, Marco M. Ferrario

**Affiliations:** Department of Medicine and Surgery, School of Medicine, University of Insubria, Viale Borri, 57, 21100 Varese, Italy

**Keywords:** Virtual reality, Simulation training, Undergraduate medical education, Medical students, COVID-19

## Abstract

**Background:**

The Coronavirus Disease 19 (COVID-19) pandemic brought significant disruption to in-hospital medical training. Virtual reality simulating the clinical environment has the potential to overcome this issue and can be particularly useful to supplement the traditional in-hospital medical training during the COVID-19 pandemic, when hospital access is banned for medical students. The aim of this study was to assess medical students’ perception on fully online training including simulated clinical scenarios during COVID-19 pandemic.

**Methods:**

From May to July 2020 when in-hospital training was not possible, 122 students attending the sixth year of the course of Medicine and Surgery underwent online training sessions including an online platform with simulated clinical scenarios (Body Interact™) of 21 patient-based cases. Each session focused on one case, lasted 2 h and was divided into three different parts: introduction, virtual patient-based training, and debriefing. In the same period, adjunctive online training with formal presentation and discussion of clinical cases was also given. At the completion of training, a survey was performed, and students filled in a 12-item anonymous questionnaire on a voluntary basis to rate the training quality. Results were reported as percentages or with numeric ratings from 1 to 4. Due to the study design, no sample size was calculated.

**Results:**

One hundred and fifteen students (94%) completed the questionnaire: 104 (90%) gave positive evaluation to virtual reality training and 107 (93%) appreciated the format in which online training was structured. The majority of participants considered the platform of virtual reality training realistic for the initial clinical assessment (77%), diagnostic activity (94%), and treatment options (81%). Furthermore, 97 (84%) considered the future use of this virtual reality training useful in addition to the apprenticeship at patient’s bedside. Finally, 32 (28%) participants found the online access difficult due to technical issues.

**Conclusions:**

During the COVID-19 pandemic, online medical training including simulated clinical scenarios avoided training interruption and the majority of participant students gave a positive response on the perceived quality of this training modality. During this time frame, a non-negligible proportion of students experienced difficulties in online access to this virtual reality platform.

## Background

Virtual reality refers to all computer technologies promoting generation of virtual environments allowing the user to interact with them [1]. In the field of undergraduate medical training, virtual reality proved to play a key role in fostering surgical skills [2–4] and to improve medical knowledge. Specifically, virtual environments proved useful to teach gross anatomy [5, 6], radiation oncology [7], endotracheal intubation [8], and, finally, to warrant proper interpretation of radiological images by medical students [9]. These studies also reported that students greatly appreciated these virtual modalities of medical training [5, 7, 9]. Over the last decade, virtual patient simulation platforms have been increasingly developed and proved helpful to further increase medical students’ ability to gather information during collection of medical history [10] and to improve their diagnostic problem-solving skills. These observations provide interesting insights when distant medical learning is necessary.

Since Coronavirus Disease 19 (COVID-19) outbreak, strict rules of social distancing have been applied worldwide [11] leading to a clearly negative strong impact on medical training with interruption of in-hospital clinical activities [12–14]. Indeed, infected students may spread the virus if asymptomatic or, viceversa, be infected during training and contribute to virus transmission [14]. In 2020 in our nation, interruption of traditional in-hospital training represents a particularly critical issue for medical students attending the last year of the course in Medicine and Surgery, who have to complete their training before graduation to apply for post-graduate residency programmes and to avoid delays in their career. Therefore, in replacement of the traditional apprenticeship at the patient’s bedside and to address the difficulties in changing medical training modality in an extremely short period of time during COVID-19 pandemic, the use of virtual reality was promptly adopted in our School of Medicine to overcome this educational gap. Although a variety of virtual teaching methods have been recently proposed [15, 16] and medical students’ appreciation for virtual teaching integrated with augmented reality platforms has been sparingly reported during COVID-19 pandemic [17], no study has reported so far the perception of medical students attending the last year of course on a fully online training including patient-based virtual reality in replacement of the traditional training at the patient’s bedside since the COVID-19 outbreak.

The aim of this study was to assess the students’ perception on out-of-hospital fully online pre-graduation training including patient-based simulation in a group of sixth-year medical students when access to conventional in-hospital training was not allowed due to COVID-19 pandemic.

## Methods

### Participants and period of training

Due to COVID-19 outbreak and the rules of social distancing worldwide, traditional in-hospital medical training was interrupted at our institution at the end of February 2020 up to the end of July 2020. Traditional training is defined as the usual in-hospital apprenticeship with tutor’s tuition at the patient’s bedside. This interruption could have had a strong impact on the career of 122 medical students all attending the sixth year of the course in Medicine and Surgery, who had to complete their training before graduation in July and early September 2020 to qualify for application for post-graduate residency programmes in late September. Therefore, they were offered to complete their practical training online including the access to a virtual reality platform (Body Interact™ Clinical Education, TakeTheWind, Coimbra, Portugal) with a variety of clinical case-based scenarios of different types and complexity.

A first and a second round of fully online training was given from early May to early June and from the end of June to the end of July for students who were expected to graduate in July and September, respectively. Considering that training was completely online, in each round of training students were split into three groups with almost equal number of students, varying from 18 to 23, to warrant the best modality of online training and to allow enough interactivity with tutors. For each group of students, 21 two-hour online training sessions to present each of the 21 simulated cases selected (see over) were scheduled with an overall number of 42 h per students’ group; a given virtual case was presented to all groups by the same tutor. In the same time frame, adjunctive training with formal online presentation and discussion of clinical cases was given. In view of the anonymous participation in the questionnaire completion, no data were available regarding gender, age and/or background of study participants nor sub-analyses according to each group’s satisfaction was possible for the same reason.

The need for consent to survey participation was waived according to the internal regulation of the School of Medicine of the University of Insubria, considering that no human data were collected and that participation was anonymous and voluntary as an extended part of routine monitoring of the quality of didactical activities.

### Body interact™ platform and training sessions

Body Interact™ is a platform which provides education training by means of virtual patients built with artificial intelligence. The software allows students to enter different simulated clinical scenarios and interact with the icon of a male or female patient to collect a thorough clinical history by interviewing him/her, perform physical examination, call for laboratory and imaging tests, administer medication, and, finally, provide interventions if necessary. Physical examination of thorax, abdomen, and peripheral pulses could be performed by means of dedicated commands provided by the virtual platform. After completion of each case, a timeline report providing a detailed sequence of actions taken by each individual student during the simulation is produced by the system and performance metrics are provided in accordance to updated clinical guidelines. Both are made available online to the tutor for students’ performance evaluation. Of note, every medical decision influences the patient’s outcome, and inappropriate decisions result in patient clinical deterioration or death. Case simulation is available on a dedicated multi-touch horizontal table or can be accessed online. For the peculiar conditions of this training, the online modality was only used.

Twenty-one patient-based clinical scenarios were made available on the platform and used for training of each group. In particular, 7 cardiovascular and cerebrovascular cases (diagnosis and management of myocardial infarction, atrial fibrillation, pulmonary embolism, ischemic stroke, intracranial haemorrhage, acute heart failure, and common cardiovascular risk factors), 6 trauma cases, 2 pneumological cases (asthma and chronic obstructive pulmonary disease), 2 with infective and gynaecology diseases (sepsis due to pneumonia and infective disorder in pregnancy), 2 of gastrointestinal surgery (haematemesis and acute cholecystitis), 1 nephrological case (acute kidney injury), and, finally, 1 case of hypoglycaemia. Cases were mainly presented in Italian. Each two-hour session was tutored and subdivided into three different parts: 30-min introduction by the tutor on the clinical case and the general functioning of the online access to Body Interact™, two independent 20-min sessions of practicing on the virtual patient during which each student was necessarily at home and used the virtual platform individually, and, finally, a debriefing phase lasting 50 min to critically discuss with the class and the tutor the virtual cases. During the introduction and debriefing phases, Microsoft® Teams platform was used to connect the entire group of students. At the completion of each case, a student’s performance report was promptly provided by the platform and tutors used this report for students’ evaluation and verification of students’ attendance. Students’ scores of the performance in each simulated case were not considered in the final analysis since it was beyond the scope of this study.

Tutors had adequate previous experience in conventional medical training but were completely novice in this training modality. However, each tutor had access in advance to the simulated cases he/she had to present.

### Questionnaire

Upon completion of training, students were invited to fill in a 12-item questionnaire with only one answer possible and a final free field available to better explain their response or to comment, if necessary. The 12-item questionnaire was prepared by the authors who evaluated the clarity, coherence, and relevance of items to obtain enough feedback of the perceived quality of this training modality and to plan future decisions. Participation into this survey was voluntary and the questionnaires were anonymized upon reception. A sample of the entire questionnaire is reported as an additional file (see Additional file 1). In particular, the questionnaire aimed at assessing prior experience of simulation, appreciation of the new modality of virtual training, whether the platform was easy to use and the clinical cases were realistic and suitable for medical training within the allotted time, and, finally, whether the platform could represent a good training modality for future generations of students beyond the pandemic phase. Answers to questions n. 3, 4, 5a, 6a, 7, 8, 9, and 11 were graded using a 4-point Likert rating scale. For quantitative analysis, 1 point was attributed to the answer “strongly disagree”, 2 points to “disagree”, 3 points to “agree”, and 4 points to “strongly agree”. For each of these questions an average score was calculated and a value > 3 was considered satisfactory.

Outcome measures and assessment of pre/post-test medical learning using Body Interact™ platform were beyond the scope of this study; data on this have been recently published by other authors [18].

### Statistical analysis

This study is based on a voluntary survey on the perceived quality of medical training received by these students with the described modality during the COVID-19 pandemic. Students who volunteered filled in the 12-item questionnaire on a voluntary basis. Therefore, for the study design no sampling methods were used for this research. Answers to each of the 12 questions were reported as percentages and represented in bars or pie charts. Answers to questions n. 3, 4, 5a, 6a, 7, 8, 9, and 11 were displayed as bar charts and the average rating was reported for each of these questions. Microsoft® Excel® v.365 was used as statistical software.

## Results

### Participants in the study

Out of 122 medical students, 115 (94%) completed the questionnaires. Fifty-six (48%) participants had prior experience with simulation training using a physical mannequin, 11 (10%) received prior training with some kind of virtual reality, 16 (14%) experienced both, and, finally, 32 (28%) were naïve to simulation.

### Ratings of the new modality of virtual training

**Fig. 1:**
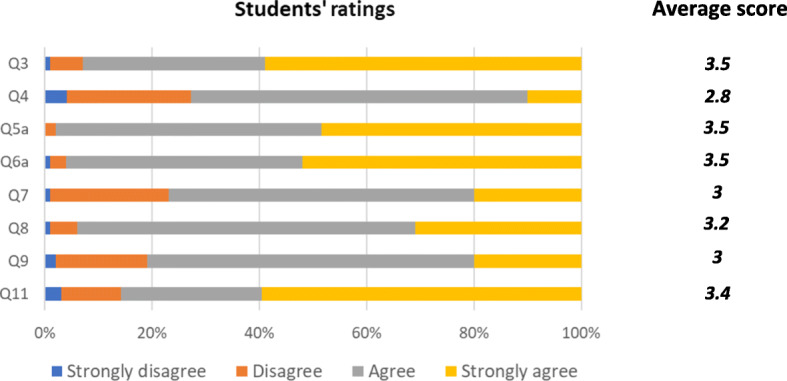
Bar chart representing medical student’s ratings on the training modality evaluated in this study. Answers to the considered questions are reported as percent values in the bars and the average score for each question is reported on the right-hand side. A score > 3 was considered satisfactory (see Text and Questionnaire model in the Additional file 1 for further details)

Compared to online formal teaching given in the same time frame, 64 (55%) students preferred online training with Body Interact™, only 8 (7%) preferred formal training, and 40 (35%) appreciated both modalities; the remaining 3 (3%) disliked both. As showed in Fig. 1, the average score calculated for questions 3 to 9 and 11 was > 3 with the only exception for question 4. Therefore, the perceived quality of this training modality investigated by these questions was considered satisfactory: these questions aimed at evaluating the format the tutored online training was structured (question 3), the type of patient-based scenarios selected (question 5), the time allotted for interacting with the clinical scenario (question 6), and whether the simulated clinical scenario was realistic and therefore useful for medical training (question 7 to 9). In fact, as investigated in the last questions, the virtual platform was considered realistic and useful by the vast majority of the participants: 89 (77%) gave a positive evaluation of the initial clinical assessment (i.e. clinical interview, physical examination), 108 (94%) of the diagnostic activity (i.e. prescription of laboratory and imaging tests), and 93 (81%) of the treatment management. Most students considered the selected cases suitable to the task (question 5) and the allotted time adequate (question 6), with 98 and 97% of positive response, respectively. Moreover, 93% of the students appreciated the three parts in which the session was split, with the time spent for interaction between the class and the tutor and the one dedicated to independent performance in the simulated case (question 3). Finally, the low rating received in question 4 (average score 2.8), which evaluated the online access to the software, is mainly explained by difficulties in accessing the platform and operating the software from remote: although 83 (72%) participants gave a positive response, 32 (28%) experienced troubles with the online access to the platform and/or technical issues with the system interface on most electronic devices, as explained in the comment section.

### Future use of virtual clinical training

**Fig. 2:**
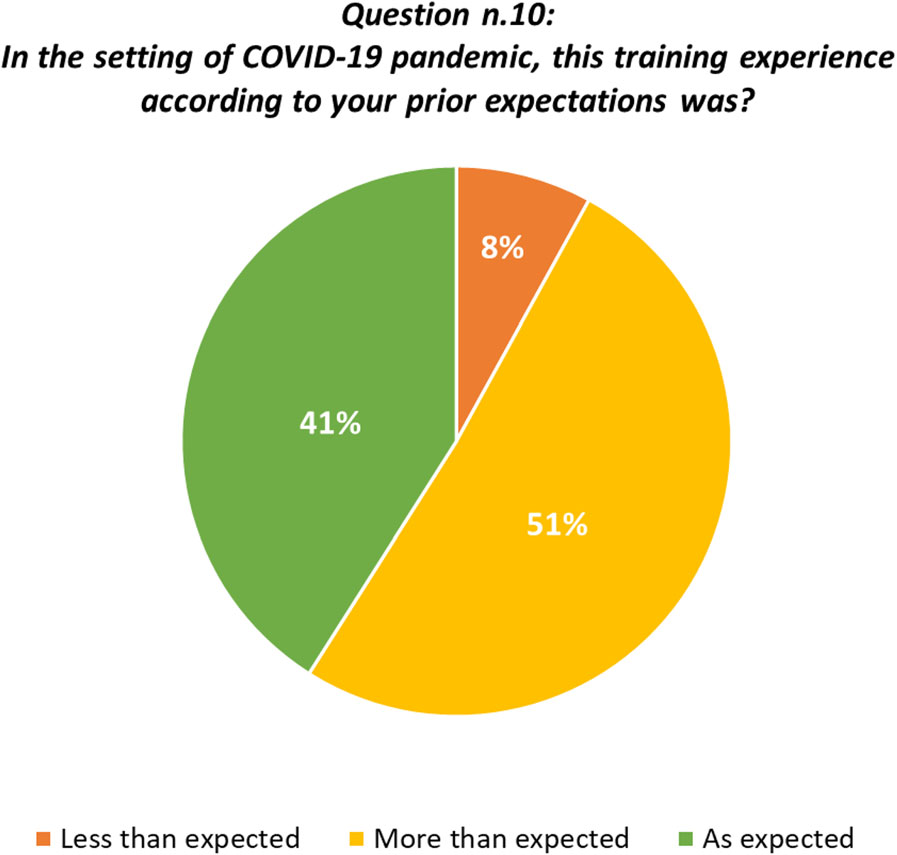
Pie chart representing how the students’ expectations were met by this training modality

**Fig. 3:**
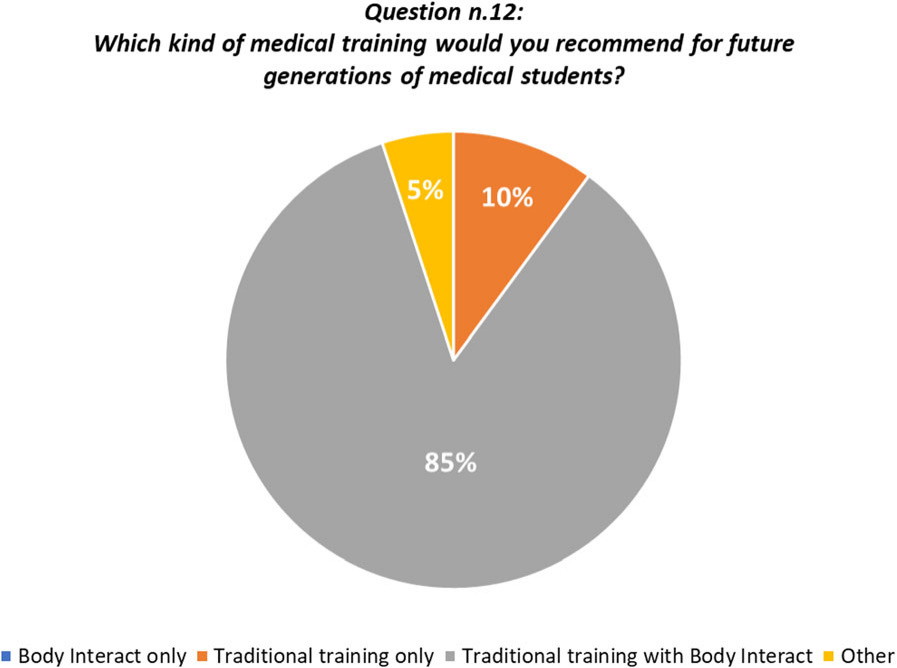
Pie chart representing which kind of training medical students suggested for future medical student generations

As showed in Fig. 2, in the setting of the COVID-19 pandemic, this new training experience met or was superior to the expectations for 92% of the participants. Moreover, as asked in question 11, 85% of the students considered this training modality useful also in the absence of potential obstacles to traditional medical training. Finally, as displayed in Fig. 3 (question 12), although most students (85%) recommended the use of Body Interact™ platform in addition to the traditional training for future generations of medical students, no one recommended the stand-alone use of this virtual reality, since the traditional medical training at the patient’s bedside and interaction with tutors was deemed invaluable by most students, as reported in the comments.

## Discussion

Medical training has been severely affected during the COVID-19 pandemic and the need to prepare future physicians has never been as crucial as it is now, in the setting of a global emergency [14]. To manage this educational need, clinical didactic sessions have been moved online with the aid of several online learning platforms [13, 14]. However, these systems could create a lack of practical and interactive experience which might have a detrimental effect on proper medical education and training among medical students [19]. Therefore, in these exceptional times, virtual simulation training could be regarded as an invaluable tool allowing students to put into practice their set of clinical skills in a completely new and innovative manner of student/tutor interaction [19]. In this new scenario, the compelling need for urgent training remodulation calls for ready-to-go platforms for out-of-hospital virtual clinical training.

Simulation-based training is emerging for teaching and learning and have proved useful in different medical fields [20–23]. New available technologies supporting complex procedures [24] are spreading worldwide and, consequently, the importance of simulation training has been increasing over the years [25, 26]. However, with the exception of some reports on nurse training [27, 28] using the Body Interact™ platform, little is known about the realism and reliability of virtual patient simulation training and no clear data are available on the level of students’ appreciation during the COVID-19 pandemic.

In this single-centre experience during the COVID-19 pandemic, a new modality of online clinical training including virtual patient reality (Body Interact™) was used instead of conventional in-hospital training, which was banned during the sanitary emergency. The format including both interaction with tutors and independent patient evaluation was greatly appreciated by most medical students and this underlines the importance of both these elements in medical training. Despite having little experience on simulators, most participants found the new simulation experience realistic (i.e. how the software presented the clinical cases in a realistic way), useful and their expectations were met. Participants highly recommended for the future generations of medical students this training modality in combination with traditional apprenticeship at the patient’s bedside, even in the absence of any potential obstacle to traditional training. Due to the online modality of using this platform, the only one possible, technical issues and troubles in the user interface with most electronic devices (i.e. desktop application and tablets) were experienced by roughly one third of the students. This is likely related to the extensive adoption of the online platform by numerous medical schools worldwide in the considered time frame. Therefore, the online modality to access this platform should be implemented to avoid delays in connecting and running a case using different devices and type of connections.

In a group of sixth-year medical students expected to graduate and apply for post-graduate courses in a short time period, these results represent the first evidence of the positive rating on a quickly employed and newly introduced online modality of virtual patient simulation training in replacement of the traditional in-hospital apprenticeship at the patient’s bedside on account of the strict rules of social distancing imposed by the COVID-19 pandemic. This should be considered in the light that the vast majority of the students and the entire group of tutors were naïve to use of this training modality, which suggests that transition to this modality can be successful even in the absence of a specific background in simulation. Differently from the results of a recently published Japanese study [18] reporting that virtual patient simulation programmes may help undergraduate medical students to improve their clinical decision-making skills even without lecturer supervision, in our study none of the participants recommended the stand alone use of virtual patient simulation for the training of future generations of undergraduate medical students. This suggests the perceived importance of the role of an experienced tutor and of traditional training in medical education, even when virtual reality is available.

Moreover, the adoption of this training modality during COVID-19 pandemic was a decision taken in a short period of time to meet the students’ need and avoid delays in their career. Evaluation of students’ scores to appraise the participant’s improvement in clinical reasoning skills using the Body Interact™ platform would have required a group of control and, therefore, more time to address this study endpoint. This was beyond the scope of this manuscript. In this regard, the usefulness of virtual patient simulation training has been recently assessed in a non-randomized study [18].

In this paper, no further evaluation was performed regarding tutors’ personal experience and the potential opportunities connected with the extensive use of this modality of virtual patient simulation at our center. Furthermore, putting together terms with different meanings, such as “useful” and “realistic”, might have introduced a potentially biased estimate of the results inferred from questions n. 7, 8, and 9. These aspects should be included among the limitations of this manuscript.

Finally, randomized prospective studies are necessary in the future to assess the adjunctive value of this training modality along with outcome measures assessing whether medical students really improve their medical knowledge and clinical reasoning skills using this modality of virtual patient reality as compared with the traditional training.

## Conclusions

The COVID-19 pandemic brought significant disruption to medical training due to rules of social distancing. However, by means of a dedicated online platform, the adaptation of medical training with integration of simulated clinical scenarios prevented medical training from being interrupted at our centre. Furthermore, this modality of training was considered useful and met the expectations of most students attending the last year of the course in Medicine and Surgery. These data suggest that online access to these resources should be implemented for remote use also for future continuation in combination with conventional training at the patient’s bedside.

## Supplementary information

**Additional file 1.** Sample of the 12-item questionnaire assessing medical students’ feedback on a modality of virtual simulation training using BODY INTERACT platform.

## Data Availability

The datasets used and/or analysed during the current study are available from the corresponding author on reasonable request.

## Abbreviations

COVID-19: Coronavirus Disease 19
