# Supplementary material for: Pre-graduation medical training including virtual reality during COVID-19 pandemic: a report on students’ perception
Source: BMC Med Educ. 2020 Sep 25;20:332. doi: 10.1186/s12909-020-02245-8 (PMC7517753; doi:10.1186/s12909-020-02245-8)
Supplement: Supplementary file 1 — Additional file 1. Sample of the 12-item questionnaire assessing medical students’ feedback on a modality of virtual simulation training using BODY INTERACT platform. [file 12909_2020_2245_MOESM1_ESM.pdf]

Additional material 1. Sample of the 12-point questionnaire assessing medical students' feedback on a new modality of virtual training using BODY INTERACT platform

1. Have you ever had simulation training experience before?

- Virtual reality only (e.g Body Interact)

- Simulation on dummy patient.

- Both

- None

2. Which of the following parts of the training did you prefer?

- Clinical case presented using slide set only.

- Clinical case presented using Body Interact

- Both

- None

3. Did you deem useful to have split this modality of virtual training into three different parts, as follows: introduction to the case, simulation training on Body Interact, and critical assessment of the case?

- Strongly disagree

- Disagree

- Agree

- Strongly agree

4. Did you find the software easy to use? (i.e. user-friendly interface, easy access to any option provided by the software to clinically manage the patient, etc.)

- Strongly disagree

- Disagree

- Agree

- Strongly agree

5*a*. Were the selected clinical cases adequate to the task?

- Strongly disagree

- Disagree

- Agree

- Strongly agree

5*b*. You would have preferred:

-More straightforward cases

-More complex cases

-The clinical cases were adequate

6*a*. Was the time allotted to each case adequate?

- Strongly disagree

- Disagree

- Agree

- Strongly agree

6*b*. You would have preferred:

-More time to complete each case

-Less time to complete each case

-Time allotted to each case was adequate

7. Did you find useful and realistic the <u>clinical history and physical examination features</u> provided by Body Interact for each case?

- Strongly disagree
- Disagree
- Agree
- Strongly agree

8. Did you find useful and realistic the <u>diagnostic activity features</u> (prescription of lab and imaging tests, etc) provided by Body Interact for each case?

- Strongly disagree
- Disagree
- Agree
- Strongly agree

9. Did you find useful and realistic the <u>clinical management and treatment options</u> provided by Body Interact for each case?

- Strongly disagree
- Disagree
- Agree
- Strongly agree

10. In the setting of COVID-19 pandemic, this training experience according to your prior expectations was?

-Less than expected

-More than expected

-As expected

11. Would you deem useful to use Body Interact virtual reality even in the absence of potential obstacles to a more traditional training (e.g COVID-19 pandemic)?

- Strongly disagree
- Disagree
- Agree
- Strongly agree

12. Which kind of medical training would you consider useful for future generations of medical students?

- Body Interact only
- Traditional training only
- Traditional training with Body Interact
- Other (specify)___________________

FURTHER COMMENTS: ____________________________________
